# Comparison of Thigh Pain in Short Versus Long Proximal Femoral Nails in Patients With Intertrochanteric Femur Fracture: A Comparative Study

**DOI:** 10.7759/cureus.78019

**Published:** 2025-01-26

**Authors:** Manu Gautam, Hitesh Garg, Aruddha Sarkar, Abhishek Sengupta, Rabi R Prasad

**Affiliations:** 1 Orthopedics, MAX Super Specialty Hospital, New Delhi, IND; 2 Orthopedics, Vardhman Mahavir Medical College (VMMC) and Safdarjung Hospital, New Delhi, IND; 3 Orthopedics, Kalinga Hospital, Bhubaneswar, IND; 4 Orthopedics and Trauma, Safdarjung Hospital, New Delhi, IND

**Keywords:** anterior thigh pain, functional outcome, intetrochanteric fracture, long pfn, short pfn

## Abstract

Introduction

Hip fractures, particularly intertrochanteric femur fractures, pose a significant public health challenge, with the global incidence projected to rise. In India, the annual incidence of osteoporotic hip fractures is expected to increase due to the growing geriatric population. The choice of fixation for these fractures remains contentious, with proximal femoral nail (PFN) emerging as a preferred option due to its biomechanical advantages. This study evaluates the outcomes of long and short PFN in managing intertrochanteric fractures, focusing on anterior thigh pain and functional outcomes.

Method

A retrospective study was conducted on 100 patients treated with long PFN (n=50) or short PFN (n=50) at MAX Super Specialty Hospital, New Delhi, from January 2020 to December 2021. Data from medical records and radiographs were analyzed for fracture union, anterior thigh pain (Visual Analog Scale (VAS) score, Verbal Rating Scale), and functional outcomes (Harris Hip Score). Complications were also assessed. Statistical analyses were performed using SPSS v23 (IBM Corp, Armonk, NY), with significance at p<0.05.

Result

The incidence of anterior thigh pain was significantly higher in the short PFN group (18%) compared to the long PFN group (2%) (p=0.017). The mean VAS score was 2.26 ± 1.42 for the short PFN group versus 1.68 ± 0.91 for the long PFN group. While the Harris Hip Scores were comparable (short PFN: 76.18 ± 11.74, long PFN: 78 ± 11.52, p=0.436), complications such as femoral canal impingement (n=4) and varus collapse (n=3) were observed exclusively in the short PFN group.

Conclusion

Long PFN demonstrated advantages in reducing anterior thigh pain and minimizing complications, particularly in populations with shorter stature and bowed femurs, common in the Indian subcontinent. Although functional outcomes were similar for both groups, long PFN appears to be the preferred choice for intertrochanteric fractures in this demographic. Further studies with longer follow-up durations are recommended to validate these findings.

## Introduction

Among one of the many important existing public health problems is the fractures of the hip. Hip treatment consumes a major fraction of healthcare expenditure. There has been two-three times increase in the incidence of hip fractures, particularly in Asia. It has been estimated to rise from 1.66 million in 1990 to 6.26 million in 2050 [1]. In 2004, a report on India estimated the annual incidence of approximately 0.6 million osteoporotic hip fractures, which is expected to increase significantly by 2026, due to improvement in the life expectancy of the geriatric population [2]. The frequency of hip fractures is increasing with age and the physically active population. Proximal femur fractures are a common group of injuries with a bimodal age distribution in elderly patients as a result of low-energy trauma or in younger patients as a result of high-energy trauma. The trochanteric fractures are constantly on the rise because of the increasing number of senior citizens with osteoporosis [3,4]. Intertrochanteric femur fractures account for 45% to 50% of fractures around the hip joint in elderly patients [5].

Recent data from the National Hip Fracture Database (NHFD) for the year 2023 indicate a 7% increase in the incidence of hip fractures, rising to 72,160 cases from an approximate range of 66,000 to 67,000 cases reported in the year 2020 and the pre-pandemic era. This upward trajectory is projected to persist, owing to demographic shifts characterized by increased life expectancy and an escalating prevalence of frailty and medical complexity within the population [6].

There are various modalities available for the internal fixation of these fractures. These fractures can be successfully treated with various types of fixations-intra-medullary or extra-medullary devices, sliding hip screws, blade plates, and cephalo-medullary nails (CMN) [7,8]. However, there is yet to decide upon the ideal implant for fixation of these fractures with different surgeons preferring different implants. The proximal femoral nail has emerged as a favored implant among surgeons given its better biomechanical properties. This intramedullary device along with providing more stability with a shorter lever arm, also shares more loading force and has less chance of collapsing after fixation of intertrochanteric femoral fractures.

While biomechanically advantageous in many ways, their use has not been without complications. There has been debate in the literature as to the optimal length of intramedullary nails. Short nails offer the advantages of shorter operative times, reduced blood loss, and lower transfusion rates. Conversely, long nails offer the theoretical benefit of protecting the full length of the femur, particularly in elderly patients with osteoporotic or osteopenic bones, thus potentially decreasing the secondary femoral shaft re-fracture rate.

An important and commonly occurring post-operative complaint of patients surgically managed with CMNs is anterior thigh pain. We believe that the use of long nails with or without a distal lock will reduce the incidence of thigh pain without affecting or changing the outcome of the fracture union.

## Materials and methods

A retrospective study was performed in the Department of Orthopedic Surgery, MAX Super Specialty Hospital, Shalimar Bag, New Delhi. All patients operated over two years with a minimum of six months follow-up from January 2020 to December 2021 were included in the study. All patients of more than 16 years of age presenting to our emergency with intertrochanteric fracture managed using Proximal femoral Nailing with radiographically confirmed union with a minimum follow-up of six months were included in the study. Patients having compound fractures, neglected inter-trochanteric fractures, fracture non-union, fracture malunion, pathological fractures, facture greater than three weeks old, polytrauma patients and patients not consenting to the study were excluded from the study.

Patients were either operated using long or short PFN based on surgeon preference. A total of 113 patients were operated during the period but only 100 patients were on regular follow up with us. Of these, 50 patients were in the long PFN group, and 50 patients were in the short PFN group.

Medical records of these patients were analyzed, and demographic data and surgical details were retrieved. Radiographs at the presentation and six-month follow-up were retrieved for all the patients. Radiographs were assessed for Union; Femoral canal impingement; Varus collapse; Cut through of proximal screws; Screw penetration and Screw back out. Patients were followed up in the outpatient department at intervals of two weeks, six weeks, three months, and six months. At the six-month visit, patients were evaluated for anterior thigh pain using the Visual Analog Scale (VAS) score and verbal rating scale. The functional status of the patients was evaluated using the Harris Hip Score (HHS). The primary outcome of our study was to analyze for anterior thigh pain in patients operated using long and short PFN and secondarily implant complications were looked at to identify any association with the same.

Data were entered into an Excel spreadsheet and analyzed using SPSS v23 (IBM Corp., Armonk, NY). Descriptive data were reported for each variable. Summarized data were presented using tables and graphs. Data were normally distributed as tested using the Shapiro-Wilk W test (p-value was less than 0.05). Mann-Whitney U test was used for the comparison of VAS scores and hip scores. The chi-square test was used for categorical variables. A level of p<0.05 was considered statistically significant. All patients were included in the study after informed written consent and proper ethical clearance was taken before pursuing the study (IEC BHR/TS/MSSH/MHIL/SHB/MHEC/ORTHO/21-08).

## Results

A total of 100 patients were included in the study with 50 patients in the long and 50 in the short PFN group. There were 23 male patients in the short PFN group and 25 male patients in the long PFN group. The mean age of the patients was 58.9±8.12 in the short PFN group and 57.9±8.94 in the long PFN group. Both groups contained patients including all three fracture types according to AO Foundation and the Orthopedic Trauma Association (AO/OTA) classification with the short PFN group having most of the 31A1.2 type and the long PFN group having 31A1.3 type (highest); however, the difference was non-significant (Table 1). As expected, the operative time for the short PFN group was significantly shorter than the long PFN group (Table 2).

**Table 1:** Distribution of participants based on AO/OTA classification Chi-square test, level of significance set at p < 0.05 Pearson chi2 (2) = 1.4693 SPFN: short proximal femoral nail, LPFN: long proximal femoral nail, AO/OTA: AO Foundation and the Orthopedic Trauma Association

Group		Preop fracture classification			Total
		31A1.1	31A1.2	31A1.3	
SPFN	N	7	23	20	50
	%	14	46	40	100
LPFN	N	6	18	26	50
	%	12	36	52	100
Total	N	13	41	46	100
	%	13	41	46	100
	P-value	0.48			

The incidence of anterior thigh pain in the short PFN group was significantly more than in the long PFN group with nine patients having VAS scores for pain more than 3 in the short PFN group and only one having VAS score greater than 3 in the long PFN group. In the Verbal rating scale, 41 patients in the short PFN group had mild pain while nine patients had moderate pain while in the long PFN group, five patients had no pain, 44 with mild pain, and only one with moderate pain.

**Table 2:** Comparison of operative time between long and short PFN Independent t-test, level of significance set at p < 0.05 SPFN: short proximal femoral nail, LPFN: long proximal femoral nail

	Mean	SD	Median	P-value
SPFN	39.26	7.714	38.4	0.0001
LPFN	55.14	9.467	57	

The HHS for the short PFN group was 76.18±11.74 while for the long PFN group, it was 78±11.52 which was found to be non-significant (p=0.436). As for complications, there were four cases of femoral canal impingement in the short PFN group and none in the long PFN group, while varus collapse was seen in three patients in the short PFN group and only one in the long PFN group. There was no incidence of proximal screw cut through or peri-prosthetic fracture in the groups while screw penetration occurred in one patient of the short PFN group and screw back out was the most common complication with 18 cases in the short PFN group and 14 in the long PFN group; however, none were statistically significant (Table 3).

**Table 3:** Distribution of complication on radiography at six months Chi square test, level of significance set at p < 0.05 Pearson chi2(1) for Femoral canal impingement = 4.1667, Pearson chi2(1) for varus collapse = 1.0417, Pearson chi2(1) for screw penetration = 1.0101, Pearson chi2(1) for screw backout = 0.7353 SPFN: short proximal femoral nail, LPFN: long proximal femoral nail

Complication	SPFN	LPFN	P-value
	N = 50	N = 50	
Femoral canal impingement	4	0	0.041
Varus collapse	3	1	0.307
Cut through of proximal screws	0	0	NA
Screw penetration	1	0	0.315
Screw back out	18	14	0.391
Peri-prosthetic fracture	0	0	NA

## Discussion

Intertrochanteric fractures are becoming quite frequent given the increasing longevity and an increasing burden of osteoporosis in the elderly. PFN has been the preferred implant of many orthopedic surgeons. PFN A was introduced in 2004 by the AO/OTA group. However, soon it was observed that the Asian population had different needs due to short stature and increased femoral anterior bow caused by increasing varus and osteoporosis commonly observed in the Asian old. This led to the development of the PFN A2 keeping in mind the special needs of the Asian populace. However, still short PFNA2 implants though come in varying sizes from 170 mm to 240 mm [9] are mainly straight and lack an anterior bow [10].

There has been an ongoing debate about whether a longer nail is better or a shorter one. Short nails require less operative time and have significantly less blood loss. Similar findings were observed in our study and have also been observed by Krishnan et al. [11] and Li et al. [12]. We operated on a total of 100 patients with 50 patients in each group. The mean age of the patients in the short and long PFN groups were 58.9+_8.12 and 57.9+_8.94, respectively, with no significant difference though our population was on the younger side compared to the groups evaluated by Krishnan et al. [11] and Li et al. [12].

Our study found that 18% of patients in the short PFN group had a VAS score of more than 3 while in the long PFN group, it was observed in only 2% of the population while 98% had a pain score of less than 3. The mean VAS score of the short PFN group was 2.26 ± 1.42, while the long PFN group had a mean VAS score of 1.68 ± 0.91 (p=0.017), which was significantly less. This is in agreement with that of Rao et al. [13], Parmar et al. [14], and Krishnan et al. [11] who found similar findings in their study. Also, Li et al. [12] mentioned in their study that thigh pains are induced by doing gamma nail fixation, which was considered to be relative to the squeeze of the nail to the femoral cortex or the irritation of the iliotibial. They further stated that three patients in whom long nails were placed can relieve themselves and it will not affect their routine life. In total, thirteen cases of thigh pain were induced by using the short nails. From 11 patients' intramedullary nails were removed to relieve pain, and medication was used for the remaining two.

Hwang et al. [15] favored long over the shorter PFNA nail especially when there is excessive anterior curvature of the femur, as there is a geometrical mismatch between the ante curvature of the femur and the contemporary intramedullary nails, which causes technical difficulties. Mukherjee et al. [10] observed that the short PFN nails introduced in India lacked curvature and had a mismatch in the nail curvature and the femoral curvature. This caused an abutment of the nail tip with the anterior cortex resulting in anterior thigh pain. They evaluated 80 patients operated using various sizes of short PFN nails, it was observed that patients with a nail tip position of 0-2 had less anterior thigh pain compared to those with a nail tip position of 3-5 (p=0.001). It was also observed that patients operated on with a nail of thinner diameter had significantly less anterior thigh pain (p=0.01). This may be attributed to the fact that thinner nails had less abutment of the anterior cortex and were more centralized in the canal compared to thicker ones. Our study also found four cases of anterior cortical abutment all of which were in the short PFN group; however, none of them had a VAS score of greater than 3. This may be explained by the short duration of follow-up in our group.

However, it was interesting to note that in spite of patients who operated with a short PFN having significantly more anterior thigh pain, there was no difference observed between the HHS of the two groups (p=0.436). Similar findings were also observed by Krishnan et al. [11] where both groups had good to excellent outcomes. In contrast, Rao et al. [13] reported a short PFN group had a better functional outcome.

The choice of the nail is guided by surgeon preference instead of evidence, however, as our observations stand, a longer PFN would be a better choice of implant in the Indian subcontinent as the general populace has a shorter stature and more bowed femur, resulting in higher incidence of anterior cortical abutment which is considered to be a prime reason for anterior thigh pain. Though there was no incidence of peri-prosthetic fracture in our group, it may be due to two short follow-up durations. However, it has been observed that shorter nails are more prone to periprosthetic fractures and one of the prime reasons why long nails were introduced was to avoid this complication. However, further studies are necessary to validate our results.

Our study was limited by a smaller sample size, heterogenous population, and short follow up though we plan to continue to follow up with these patients to identify long-term results.

## Conclusions

We can conclude from our study that though functionally both short and long PFN nails have similar outcomes for the Indian diaspora, a long nail is a better option given its lower incidence of anterior thigh pain and lesser risk of periprosthetic fractures. However, longer nails are limited by more operative time and more blood loss. Further studies are required to find conclusive evidence on which is the best nail; however, the needs of the Indian population should also be taken into consideration before making a decision.
